# Matched Analysis of Circulating and Adipose Tissue SIRT1 Protein Level in Human Obesity

**DOI:** 10.3390/nu18081239

**Published:** 2026-04-15

**Authors:** Luisa Salvatori, Francesca Megiorni, Giorgia Maria Baldazzi, Valentina Ventimiglia, Elena Gangitano, Mikiko Watanabe, Orietta Gandini, Eleonora Poggiogalle, Lucio Gnessi, Carla Lubrano, Daniele Gianfrilli, Andrea Maria Isidori, Antonio Angeloni, Stefania Mariani

**Affiliations:** 1 Institute of Molecular Biology and Pathology, National Research Council (CNR), c/o Sapienza University of Rome, 00185 Rome, Italy; 2 Department of Well-Being, Health and Environmental Sustainability, Sapienza University of Rome, 02100 Rieti, Italy; 3 Department of Experimental Medicine, Sapienza University of Rome, 00161 Rome, Italy; 4 Department of Molecular Medicine, Sapienza University of Rome, 00161 Rome, Italy

**Keywords:** SIRT1, subcutaneous adipose tissue, plasma, obesity

## Abstract

**Background/Objectives**: Mammalian sirtuins (SIRTs) are evolutionarily conserved proteins that are epigenetically involved in biological processes such as metabolism and longevity. SIRT1 expression is reduced in metabolic disorders and in complicated diseases such as obesity. However, whether the SIRT1 level in subcutaneous adipose tissue (SAT) matches with its circulating form in obesity is unknown. The aim of our study is to evaluate SIRT1 derived from SAT and plasma of the same subject in individuals with and without obesity to assess whether plasma measurements may provide clinically significant information. **Methods**: Eleven subjects with obesity (BMI ≥ 30 kg/m^2^) and six controls without the disease (BMI < 30 kg/m^2^) were enrolled, and SIRT1 was measured in SAT and plasma by ELISA. Anthropometric parameters, glycemia and transaminases were also assessed. **Results**: Patients with obesity showed similar levels of SIRT1 in SAT and plasma (1.28 ± 0.45 and 1.9 ± 0.25 ng/mL, respectively, *p* = 0.243). Patients without obesity showed higher SIRT1 levels in SAT than in plasma (4.19 ± 1.33 and 1.06 ± 0.12 ng/mL, respectively, *p* = 0.039). An inverse correlation between SAT-derived SIRT1 and BMI was found (*r* = −0.632, *p* = 0.007). **Conclusions**: In this pilot study, our results show that the plasma SIRT1 levels substantially reflect those of SAT in patients with obesity. Given the metabolic role of SIRT1, further comprehensive investigations in larger longitudinal cohorts are needed to support plasma SIRT1 as an eligible diagnostic tool for stratifying metabolic risk associated with fat mass expansion in obesity.

## 1. Introduction

Sirtuins (SIRTs) are NAD^+^-dependent deacetylase enzymes that are ubiquitously expressed. They play a role as metabolic sensors and transcriptional regulators, linking nutrient status to energy homeostasis, mitochondrial function, inflammation and aging [1,2]. Indeed, SIRTs are emerging as targets to treat cardiac and metabolic disorders and other diseases [3].

SIRT1 is the best-known member of the family. SIRT1 transgenic mice are protected against obesity, liver steatosis, diabetes mellitus, neurodegenerative diseases, osteoporosis, etc. [1,4]. SIRT1 expression has been extensively studied across multiple tissues. In adipose tissue (AT), where it is particularly abundant, it plays a role in adipocyte differentiation, proliferation, and regulation of white fat browning [5]. SIRT1 activation promotes mitochondrial biogenesis in adipocytes and reduces AT inflammation [4]. In humans, SIRT1 has been shown to limit visceral fat accumulation by restraining adipogenesis of visceral adipose-derived stem cells (ASCs) [6]. Accordingly, SIRT1 mRNA expression is markedly lower in visceral ASCs isolated from patients with obesity compared to individuals without obesity [7,8].

An inverse correlation between the fat mass (FM) amount and SIRT1 expression in AT has been observed in several studies [9,10]. Weight gain and the concomitant SIRT1 drop worsen overall health, thus underlining the metabolic protective role played by SIRT1 against fat excess [11,12]. Interestingly, adipose-specific knockdown of SIRT1 leads to the development of obesity and insulin resistance, as mice exhibit increased FM, impaired glucose tolerance and attenuated insulin sensitivity [13]. Conversely, the reduction in FM achieved through either surgical devices or dietary calorie restriction is associated with increased SIRT1 levels in both blood [14,15] and tissues [16], leading to undoubted cardiometabolic health benefits. In line with this evidence, higher levels of circulating SIRT1 have been found in patients with a very low FM amount [11]. These clinical and experimental in vivo observations support the translational relevance of SIRT1 as a key mediator, and potential biomarker, of metabolic health in humans.

SIRT1 activity encompasses multiple cellular processes, including glucose uptake, lipid utilization, regulation of apoptosis and cell survival, feeding behavior, and hormonal release. Disruption of these processes due to SIRT1 activity reduction contributes to diminished metabolic resilience and increased disease susceptibility [13]. Furthermore, beyond metabolic dysregulation, low levels of SIRT1 have been consistently associated with clinical frailty in human models of obesity and chronic metabolic disease, supporting SIRT1 as a distinctive biomarker of frailty [1,17,18,19]. Patients with obesity frequently exhibit a frailty phenotype, characterized by reduced physiological reserve and increased vulnerability to stress, and SIRT1 downregulation is among the mechanistic links between excess adiposity, sarcopenia, metabolic dysfunction, and systemic frailty. Indeed, obesity-related complications, such as liver steatosis, type 2 diabetes, cardiomyopathy, and insulin resistance, are associated with frailty and all characterized by impaired SIRT1 activity [20,21,22].

Given the crucial role of SIRT1 both against obesity and in mitigating obesity-related complications, it is important to understand how circulating SIRT1 correlates with its expression in AT, to readily obtain clinically meaningful information. In this study, we aimed to compare matched SIRT1 measurements in plasma and subcutaneous adipose tissue (SAT) obtained from the same individual, to clarify whether plasma SIRT1 can reliably reflect SIRT1 expression in AT or depot-specific changes. By assessing the concordance between SAT-derived and circulating SIRT1 levels in obesity, our findings would strengthen the rationale for investigating plasma SIRT1 as a potential non-invasive biomarker with possible relevance to the development of therapeutic strategies for obesity.

## 2. Materials and Methods

### 2.1. Patients’ Enrolment

Eleven patients affected by obesity (body mass index, BMI ≥ 30 kg/m^2^, 5 males and 6 females, age range 35–78 years) and 6 subjects without obesity, hereinafter referred to as controls (BMI < 30 kg/m^2^, 3 males and 3 females, age range 26–68 years) were enrolled in the study. Patient recruitment occurred at the Department of Surgical Sciences, Policlinico Umberto I, Sapienza University of Rome, Italy, where they were scheduled for gallbladder stone surgery. No formal a priori power calculation was performed, and the sample size was determined by feasibility constraints related to surgical biopsy collection. The inclusion criteria were as follows: stable weight for at least 1 month before the study, needing surgical procedures, BMI ≥ 30 kg/m^2^ for patients affected by obesity and BMI between 18.5 and 29.9 kg/m^2^ for control subjects. Exclusion criteria included systemic corticosteroid therapy and all therapies that could potentially alter body weight and body composition, full-blown hypothyroidism, cirrhosis and other chronic liver diseases, diabetes, Cushing disease, and acute diseases. Among exclusion criteria based on dietary recall, we considered the use of any dietary supplements or bioactive compounds potentially affecting SIRT1 activity/concentrations; based on dietary assessment, we considered recent changes in energy intake.

Physical examination, anthropometric measurements, determination of biochemical glycemia, aspartate aminotransferase (AST), alanine aminotransferase (ALT) and plasma SIRT1 were performed after an overnight fast for all patients entering the study.

The study was approved by the Institutional Ethics Committee of Policlinico Umberto I-Rome (protocol code 5475, date of approval 24 October 2019) according to the Declaration of Helsinki, and all participants provided written informed consent for participation in the research as well as for the publication of their clinical and biochemical information.

### 2.2. Sample Collection

Blood was collected in EDTA tubes, kept on ice, and centrifuged at 2000× *g* for 10 min at 4 °C within 30 min from collection. Plasma was aliquoted and stored at −80 °C until analysis. Paired white SAT (hereinafter referred to as SAT) samples were obtained by surgical biopsy from the subcutaneous fat layer of the abdominal wall and immediately stored at −80 °C.

### 2.3. SIRT1 Assay

The SIRT1 concentration was determined both in the plasma and SAT by using the human NAD-dependent Deacetylase Sirtuin-1 ELISA Kit (MyBioSource, Cod. GDMBS705558, San Diego, CA, USA) according to the manufacturer’s instructions. Briefly, 100 mg of SAT were homogenized in 1 mL PBS and analyzed parallel to the plasma obtained from the same subject. Seven different concentrations of purified SIRT1 (0.156–0.312–0.625–1.25–2.5–5.0–10 ng/mL) were used to plot a standard curve. One hundred microliters of plasma, tissue homogenate or standards were added to each well of the microplate containing the SIRT1 antibody. After removing any unbound substances, a specific biotin-conjugated antibody followed by an avidin-conjugated Horseradish Peroxidase was added to the wells. Finally, a substrate solution was added to allow for color development that was in proportion to the amount of SIRT1 bound in the initial step. The intensity of the color was measured using a D3 Plate Reader (DAS, Italy) set to 450 nm.

### 2.4. Statistical Analysis

Data were analyzed using GraphPad Prism v5.01 (GraphPad Software, San Diego, CA, USA). The statistical significance between SIRT1 values in SAT and plasma was determined with the unpaired Student’s *t*-test. Pearson’s correlation coefficient test was used to analyze the relationships between variables. *p* values of <0.05 were considered significant.

## 3. Results

Table 1 shows the main characteristics of patients stratified into obesity and controls according to their BMI. The groups were homogeneous in age and gender. The mean BMI was 37.91 ± 2.29 kg/m^2^ in patients affected by obesity, and 25.28 ± 1.09 kg/m^2^ in the control subjects. The group differences in weight and BMI were statistically significant (*p* < 0.005). On the contrary, no significant differences in glycemia and transaminases were observed. Overall, the lack of differences in glycemia and transaminases indicates that the metabolic influence on SIRT1 expression is similar between obesity and the controls.

**Figure 1:**
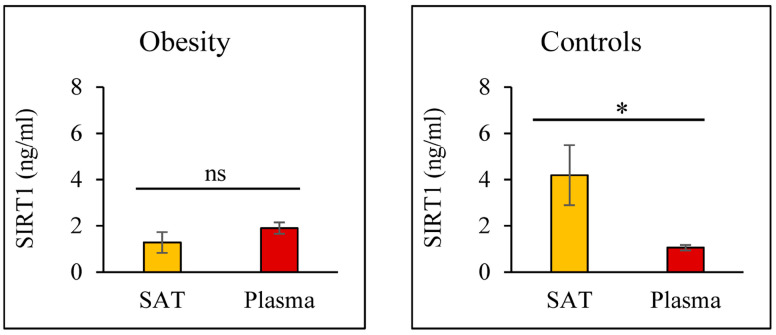
Adiposity affects the relationship between SIRT1 levels in SAT and plasma. Comparison between the mean levels of SIRT1 in SAT (yellow columns) and plasma (red columns) in patients with obesity (**left**) and controls (**right**). Data are presented as mean values ± SEM. * *p* < 0.05.

For each subject involved in the study, the SIRT1 levels were measured in SAT and plasma to compare tissue and circulating protein concentrations in both the obesity and control groups. As shown in Figure 1, patients with obesity showed similar SIRT1 concentrations in SAT and plasma samples without any statistically significant difference (1.28 ± SEM 0.45 and 1.9 ± SEM 0.25 ng/mL, respectively, *p* = 0.243). On the contrary, subjects without obesity showed significantly higher expression of SIRT1 in SAT than in plasma (4.19 ± 1.33 and 1.06 ± 0.12 ng/mL, respectively, *p* = 0.039). To note, the ratio between SAT and plasma SIRT1 was progressively more pronounced from obesity (SAT/plasma SIRT1 = 0.67) to the controls (SAT/plasma SIRT1 = 3.96), particularly in the thinner controls (overweight SAT/plasma SIRT1 = 2.02; lean SAT/plasma SIRT1 = 6.82).

**Figure 2:**
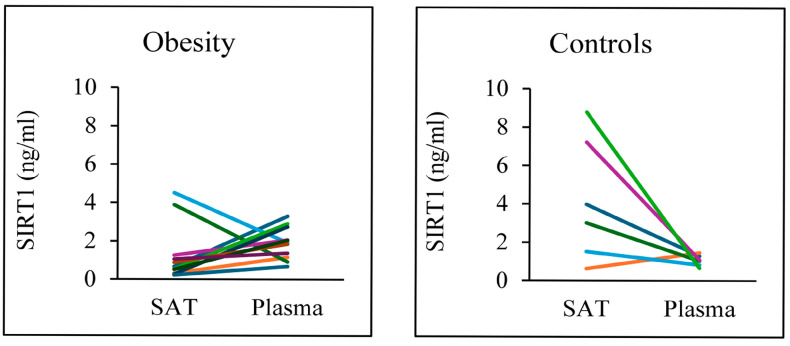
SIRT1 levels in all patients analyzed. Each line indicates the protein level in SAT and plasma in the same subject.

To better highlight individual variability in SIRT1 expression found in SAT and plasma, Figure 2 shows multi-line graphs where each line represents a subject. With the exception of two individuals, all patients with obesity (left) showed similar SIRT1 levels in both the SAT and plasma, suggesting a consistent intra-individual relationship. To note, the two patients with obesity who had higher levels of SIRT1 in SAT also had BMI equal to 30 kg/m^2^ and 30.1 kg/m^2^, indicating a borderline condition with overweight. In contrast, most of the control subjects (right), particularly five out of six, showed higher levels of SIRT1 in SAT and lower in plasma. It is noteworthy that the extension of the distribution of SIRT1 in SAT varied greatly from patient to patient, while the same amplitude was not found for the plasma levels of SIRT1 that substantially converged in a restricted zone of values. In the control subjects, the pronounced interindividual variability in SAT-derived SIRT1, in contrast to the tight clustering of plasma SIRT1 concentrations, suggests that SIRT1 expression within AT is highly heterogeneous. This heterogeneity likely reflects differences in AT health, immunologic milieu, and remodeling dynamics. Conversely, circulating SIRT1 appears to integrate inputs from multiple tissues, providing a more stable and buffered systemic measure.

**Figure 3:**
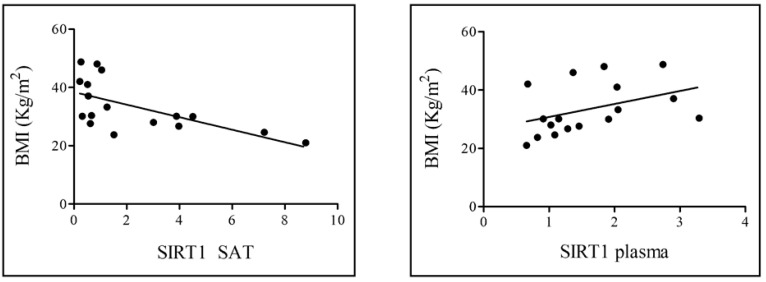
Pearson correlation analysis between SAT-derived SIRT1 and BMI (**left**), *p* = 0.007, and plasma-SIRT1 and BMI (**right**), *p* value = 0.106.

The correlation between SIRT1 and BMI was also analyzed (Figure 3). Our results showed that while SAT-derived SIRT1 was negatively related to BMI (left, *r* coefficient = −0.632), circulating SIRT1 was positively related to BMI (right, *r* coefficient = 0.406).

## 4. Discussion

During weight gain, selective remodeling events reshape human AT phenotype [23]. In this study, we observed that SIRT1 levels in SAT and plasma are substantially comparable in patients with obesity, suggesting that the circulating SIRT1 measurement mirrors that of SAT. Differently, in patients without obesity, SIRT1 shows higher levels in SAT than in plasma, presumably exercising a significant protective role locally. Such distinct behavior suggests a precise regulation of SIRT1 based on the degree of adiposity, reinforcing its role as a nutrient- and energy-sensitive molecular sensor.

The mammalian SIRTs family comprises the protein enzymes SIRT1 to SIRT7, which have huge potential to promote longevity through epigenetic activity in cell signal transduction and metabolism enhancement [24]. SIRT1 is recognized as a cellular metabolic sensor, which responds to caloric intake and FM amount. It is essential in the maintenance of systemic energy homeostasis, allowing the cell to adapt to nutrient availability [25]. Fasting or weight loss increases SIRT1 expression levels. Thus, while SIRT1 is low in obesity, the highest concentrations are observed in severely underweight patients [11].

In general, the greater the caloric intake, the greater the reduction in SIRT1 expression, and the greater the loss of control over glucose and lipid metabolism in the liver, insulin secretion in the pancreas, fat mobilization and brown remodeling in white AT [2,26]. Epigenetics is among the several factors implicated in the pathogenesis of obesity [27]. In this regard, SIRT1 plays an intricate epigenetic role in the regulation of fat metabolism by histone modifications, gene activity control, and mitochondrial biogenesis [24,28]. Worsening of these activities causes several metabolic derangements, and the largely dysfunctional AT induced by obesity determines metabolic complications to which SIRT1 activity reduction may eventually contribute. Therefore, from a pathophysiological point of view, while the AT of subjects affected by obesity is a diseased tissue with hypertrophic adipocytes and a strong inflammation in macrophages, exacerbated by lipotoxicity and lower SIRT1 production [29,30], the AT of patients without obesity is less inflamed [23] and shows greater flexibility, which is possibly supported by the increased production of locally active SIRT1 [9].

In past decades, SIRT1 expression in tissues has been intensely studied, while the evaluation of circulating protein is more recent. Indeed, it is not known if the AT and the blood SIRT1 content move in parallel, or how they are interrelated. Therefore, a novel and clinically relevant finding of our pilot study is the differential relationship between SAT and circulating SIRT1 in individuals with and without obesity. Indeed, we observed that patients with obesity have comparable values of SIRT1 in SAT and plasma, suggesting that circulating SIRT1 reliably reflects tissue expression in this condition. In contrast, SAT-derived SIRT1 levels were higher than plasma levels in subjects without obesity, indicating a dissociation between tissue abundance and circulating availability. These expression patterns support the hypothesis that plasma and tissue SIRT1 are not independent variables, but are instead biologically linked, with their relationship being modulated by the individual fat amount. The direct comparison of SIRT1 levels in SAT and blood within the same individuals adds a meaningful degree of novelty to our work, enabling a more integrated interpretation of their relationship.

In obesity, chronic caloric excess and AT dysfunction may lead to a generalized downregulation of SIRT1, resulting in similarly low levels both locally and systemically. Under these conditions, plasma SIRT1 may serve as a surrogate marker of SIRT1 content in AT, and the absence of a difference between SAT and plasma SIRT1 supports the concept that the circulating measurement mirrors the AT’s condition. So, assessing circulating SIRT1 may provide indirect information on the SIRT1 status in AT, and this observation is particularly relevant given the invasive nature of AT biopsies. From a translational perspective, plasma SIRT1 assessment may therefore represent a feasible and informative biomarker to stratify metabolic risk associated with FM expansion and AT dysfunction in obesity. Conversely, in non-obese individuals, the elevated levels of SIRT1 found in SAT compared to plasma corroborates its protective effects against metabolic stress by preserving adipocyte function and insulin sensitivity, suggesting that healthy AT retains SIRT1 intracellularly, limiting its release into the bloodstream.

Even though currently there is no definitive evidence that SIRT1 is leaked due to adipocyte damage, rather than being actively secreted or regulated via different pathways, this reasoning is conceptually analogous to what is observed for other intracellular enzymes, such as transaminases, which are released into circulation primarily in response to cellular stress or local tissue damage. Consequently, in obesity, measuring plasma SIRT1 may provide indirect yet meaningful information on SIRT1 status in AT, whereas in individuals without obesity, plasma SIRT1 appears to be less informative of tissue expression.

Although circulating SIRT1 originates from multiple organs, AT appears particularly sensitive to metabolic stress, and previous studies have shown that SIRT1 expression in obesity is more severely reduced in AT than in skeletal muscle [31]. This highlights AT as a primary contributor to metabolic SIRT1 dysregulation and reinforces the relevance of focusing on SAT as a key tissue linking obesity to systemic SIRT1 alterations. In line with these concepts, we found a significant negative correlation only between SAT-derived SIRT1 and BMI, suggesting that the increase in BMI, although a surrogate index of adiposity, corresponds to a lower SIRT1 concentration in SAT. The opposite behavior of circulating SIRT1 in relation to BMI, which is also reported in other studies [14,32,33], shows the non-univocal trend of SIRT1 in relation to measures of adiposity other than fat mass.

Recently, a number of nutrients and food components have been identified as potential activators of SIRT1 [34]. In particular, polyphenols and bioactive compounds, such as resveratrol, can interfere with SIRT1 activity, modulating SIRT1 responses other than those related to dietary energy changes. Extant data, mainly from animal studies, are promising, providing observations of beneficial metabolic effects from some food ingredients (such as curcumin, quercetin, berberine, quinine, etc., available in fruits, vegetables, and plants) at multiple levels, including oxidative stress, inflammation, and autophagy [34,35,36]. Moreover, it has been reported that central administration of resveratrol was able to mitigate derangements in glucose tolerance associated with a high-fat diet [37]. Therefore, for better metabolic health, greater attention should be paid to the consumption of and supplementation with food ingredients that are bioactive molecules in the complex interplay between SIRT1 and nutrient sensing.

A major limitation of this study is the small sample size, which is linked to the difficulty of obtaining fat samples from humans, together with the lack of a direct evaluation of the FM percentage. Moreover, the lack of data on visceral adipose tissue (VAT), which is a more metabolically active tissue, is also a significant gap. Future studies, also including VAT, should investigate SIRT1 expression in a larger cohort of individuals with and without obesity, further exploring the translational and clinical relevance of the interaction between AT and circulating SIRT1. In addition, it will be crucial to elucidate the molecular mechanisms regulating SIRT1 release into circulation and to determine how pharmacological or lifestyle interventions could modulate the SIRT1 levels in AT and plasma in order to validate SIRT1 as a circulating dynamic biomarker of prognostic value in obesity-related complications and as an indicator of therapeutic response.

## 5. Conclusions

In conclusion, our data suggest that adiposity profoundly influences SAT and circulating SIRT1’s reciprocal relationships. Since circulating SIRT1 mirrors SAT-derived SIRT1 expression in obesity, the SIRT1 level could be explored as a potential non-invasive biomarker that is capable of providing clinically relevant information on AT’s metabolic status. Given the practical limitations in assessing SIRT1 in AT directly, future more extensive studies could propose blood SIRT1, together with established diagnostic tools, to stratify the metabolic risk associated with FM expansion in obesity. In contrast, in subjects without obesity, elevated SIRT1 expression in SAT appears to represent a protective reservoir that is uncoupled from circulating levels, underscoring the importance of metabolic context when interpreting plasma SIRT1 measurements. Overall, these findings open new perspectives for the clinical application of circulating SIRT1 evaluation in metabolic risk stratification, although validation in larger longitudinal cohorts is needed. Finally, future research should be prompted to assess the effects of dietary manipulation and specific nutrient supplementations in the modulation of SIRT1 activity, to counteract SIRT1 alterations related to energy imbalance and chronic exposure to nutrient excess, paving the way for precise nutrition strategies for obesity management.

## Figures and Tables

**Table 1:** Anthropometric and biochemical characteristics of the subjects enrolled in the study.

	Obesity	Controls	*p*-Value
N. subjects	11	6	-
Gender (male/female)	5/6	3/3	-
Age (years)	52.45 ± 4.36	55.83 ± 6.48	0.662
Weight (kg)	107.2 ± 8.87	69.5 ± 2.39	0.008
BMI ^1^ (kg/m^2^)	37.91 ± 2.29	25.28 ± 1.09	0.0015
Glycemia (mg/dL)	93.51 ± 2.46	90.08 ± 3.53	0.426
AST ^2^ (U/L)	26.71 ± 9.9	28.33 ± 10.1	0.911
ALT ^3^ (U/L)	33.13 ± 9.63	24.83 ± 8.84	0.552

^1^ BMI, body mass index. ^2^ AST, aspartate aminotransferase. ^3^ ALT, alanine aminotransferase.

## Data Availability

The original contributions presented in this study are included in the article. Further inquiries can be directed to the corresponding author.

## Abbreviations

The following abbreviations are used in this manuscript:

SIRTs	Sirtuins
AT	Adipose tissue
ASCs	Adipose-derived stem cells
FM	Fat mass
SAT	Subcutaneous adipose tissue
BMI	Body mass index
AST	Aspartate aminotransferase
ALT	Alanine aminotransferase
